# Is *prnt* a Pseudogene? Identification of Ram Prt in Testis and Ejaculated Spermatozoa

**DOI:** 10.1371/journal.pone.0042957

**Published:** 2012-08-24

**Authors:** Jorge Pimenta, Ana Domingos, Pedro Santos, Carla C. Marques, Cátia Cantante, Ana Santos, João P. Barbas, Maria C. Baptista, António E. M. Horta, Aldino Viegas, Patrícia Mesquita, João Gonçalves, Carlos A. Fontes, José A. M. Prates, Rosa M. L. N. Pereira

**Affiliations:** 1 Unidade de Recursos Genéticos, Reprodução e Melhoramento Animal, Instituto Nacional dos Recursos Biológicos (INRB) L-INIA Santarém, Quinta da Fonte Boa, Vale de Santarém, Portugal; 2 CIISA, Faculdade de Medicina Veterinária (FMV), Universidade Técnica de Lisboa, Lisboa, Portugal; 3 IHMT-CMDT – Instituto de Higiene e Medicina Tropical, Centro de Malária e Doenças Tropicais, Lisboa, Portugal; 4 Hospital Universitário de Coimbra, Coimbra, Portugal; 5 Unidade de Retrovírus e Infecções Associadas (URIA), ADEIM-Centro de Patogénese Molecular/Instituto de Medicina Molecular (IMM), Faculdade de Farmácia, Universidade de Lisboa, Lisboa, Portugal; 6 REQUIMTE/CQFB Departamento de Química, Faculdade de Ciências e Tecnologia, Universidade Nova de Lisboa, Caparica, Portugal; 7 Escola Universitária Vasco da Gama, Coimbra, Portugal; Ecole Normale Superieure de Lyon, France

## Abstract

A hallmark of prion diseases or transmissible spongiform encephalopaties is the conversion of the cellular prion protein (PrP^C^), expressed by the prion gene (*prnp*), into an abnormally folded isoform (PrP^Sc^) with amyloid-like features that causes scrapie in sheep among other diseases. *prnp* together with *prnd* (which encodes a prion-like protein designated as Doppel), and *prnt* (that encodes the prion protein testis specific - Prt) with *sprn* (shadow of prion protein gene, that encodes Shadoo or Sho) genes, constitute the “prion gene complex”. Whereas a role for *prnd* in the proper functioning of male reproductive system has been confirmed, the function of *prnt*, a recently discovered prion family gene, comprises a conundrum leading to the assumption that ruminant *prnt* is a pseudogene with no protein expression. The main objective of the present study was to identify Prt localization in the ram reproductive system and simultaneously to elucidate if ovine *prnt* gene is transcribed into protein-coding RNA. Moreover, as Prt is a *prnp*-related protein, the amyloid propensity was also tested for ovine and caprine Prt. Recombinant Prt was used to immunize BALB/c mice, and the anti-Prt polyclonal antibody (APPA) immune response was evaluated by ELISA and Western Blot. When tested by indirect immunofluorescence, APPA showed high avidity to the ram sperm head apical ridge subdomain, before and after induced capacitation, but did not show the same behavior against goat spermatozoa, suggesting high antibody specificity against ovine-Prt. Prt was also found in the testis when assayed by immunohistochemistry during ram spermatogenesis, where spermatogonia, spermatocytes, spermatids and spermatozoa, stained positive. These observations strongly suggest ovine *prnt* to be a translated protein-coding gene, pointing to a role for Prt protein in the ram reproductive physiology. Besides, caprine Prt appears to exhibit a higher amyloid propensity than ovine Prt, mostly associated with its phenylalanine residue.

## Introduction

Prion gene family comprises four identified genes: the prion protein gene, *prnp*, the prion-like protein gene, *prnd*, the shadow of prion protein gene, *sprn* and the prion protein testis-specific gene *prnt* [1]. *prnp*, and its homologues, *sprn*, *prnd* and *prnt*, show similar gene organizations, which encompass two or three exons [2]. Mammalian *prnp*, that encodes the cellular prion protein (PrP^C^), is a housekeeping gene, present in both eutherians and fish [3] that has been characterized in several species, like hamster [4], human, sheep, mouse [5] and bovine [6]. *prnd*, which encodes a prion-like protein designated as Doppel (or Dpl) is located 16–52 kb downstream from *prnp*, depending on the species [7]–[10]. *prnd* contributes together with *prnp*, the recently discovered *prnt*, that encodes the prion protein testis specific – Prt [8], and the *sprn* shadow of prion protein gene, that encodes Shadoo [3], to the so called “prion gene complex”. Although the normal physiological function of the cellular prion protein, PrP^C^, remains largely unclear, an aberrant conformation with amyloid-like features of PrP^C^ is thought to be the essential component of the infectious particle, involved in transmissible spongiform encephalopaties (TSE) in mammals [11]. The normal prion protein exists in the α-helix-rich form and is harmless while the pathogenic form exists as rod-like particles with 10–12 nm in diameter. Besides TSE, these rod-like particles known as amyloids, are associated to a large number of diseases such as Alzheimer's disease, diabetes mellitus type 2, Parkinson's disease, among many others [12], [13]. *prnp* is a single-copy gene, but other *prnp*-related proteins, may also contribute to the pathogenesis of TSEs [7] demanding further investigation.

Several studies reported that *prnd* as well as *prnt* are highly expressed in the testicular tissue [7], [9], [14], which allied to the sterility of *prnd* ^0/0^ mice [15], [16] and the enhanced fertilizing ability of ovine spermatozoa (spz) after Doppel supplementation [17], suggest an important physiological role on male fertility. Prion protein testis-specific gene, *prnt*, was described as being closer to *prnd* than *prnp* in the human genomic sequence. *prnt* is located 3 kb in human [7], and 6 kb in cattle [18], 3′ downstream of *prnd*, and possibly emerged due to a duplication event that occurred early during eutherian species divergence [7], [19]. This gene has a two-exon structure in humans [7]. Likely two-exon RNA structures that align to >90% of human *prnt* can also be predicted in cow, sheep, horse and dog [19]. Vertebrate CpG islands (CGIs) are short interspersed DNA sequences that deviate significantly from the average genomic pattern by being GC-rich, CpG-rich, and predominantly non-methylated. Most, perhaps all, CGIs are sites of transcription initiation, including thousands that are remote from currently annotated promoters [20]. Housekeeping genes use primarily CpG-dependent core promoters, whereas the majority of tissue-specific genes possess neither CGIs nor TATA-boxes in their core promoters [21]. While the eutherian *prnp* and *sprn* promoters incorporate CGIs, the *prnd* and *prnt* promoters do not include CGIs [2], [7] suggesting that these genes are expressed in a tissue-specific manner. Analysis of both adult and fetal human tissues confirmed the ubiquitous but variable expression profile of *prnp*, with the highest levels observed in the Central Nervous System and testis. Contrastingly, *prnd* shows a wide tissue expression pattern in fetal tissues, while it is expressed exclusively in adult testis implying that *prnd* is developmentally regulated. In addition, all three human *prnt* isoforms were detected only in adult testis [7]. In caprine, *prnt* is weakly and stochastically expressed in both testes and ovaries at various development stages, suggesting either that the expression pattern of this gene differs between ruminant and human or, most probably, that ruminant *prnt* is a pseudogene [18]. Some pseudogenes appear to harbor the potential to regulate their protein-coding cousins. Far from being silent relics, many pseudogenes are transcribed into RNA, some exhibiting a tissue-specific pattern of activation [22]. The caprine and bovine genomes contain a 227-bp-long *prnt* region, showing 100% identity, both with a premature stop codon, and a second AUG initiation codon located downstream [18]. Homology between human PrP^C^ and Prt is lower (44% similarity and 30% identity) than between ORFs (open reading frames) from *prnd* and *prnt* (50% similarity and 42% identity). In bovine, *prnt* encodes for an N-terminally truncated protein of 55 amino acids (aa) in length, homologous (55% identity) to its human counterpart [18]. In humans, the potential ORF for *prnt* has 94 aa before a TAA stop codon [7]. No signal peptides were predicted for Prts, which suggests that Prts could be intracellular proteins [2]. Prt distribution in caprine and human tissues was studied, so far, only at mRNA level [7], [18]. The tissue distribution and localization of this protein, in each tissue, has not yet been investigated. The main aim of this work was to identify Prt localization in ram spz and testicular tissue, confirming that ovine *prnt* gene is transcribed into protein-coding RNA. Furthermore, and although Prt involvement in TSEs has not yet been confirmed, we also tested for ovine and caprine Prt amyloid propensity as Prt is a prnp-related protein.

## Materials and Methods

This experimental protocol was reviewed by the Ethics Committee of CIISA/FMV and approved by the Animal Care Committee of the National Veterinary Authority (Direcção-Geral de Veterinária, Portugal), following the appropriate European Union guidelines (N. 86/609/EEC).

### 1. Immunization of animals

The ovine 52 aa Prt peptide (Accession number ABO86196.1) was obtained from CASLO Laboratory ApS (Denmark) showing 96.88% purity, and used without further purification. Five to six-week-old female BALB/c mice were injected intraperitoneally with 20 µg of purified ovine Prt emulsified in incomplete Freund's adjuvant (Sigma-Aldrich, St. Louis, USA) and boosted monthly (four times), with an equal mass of ovine Prt.

### 2. Enzyme linked immunosorbent assay (ELISA)

Antibody responses generated against ovine Prt were measured by ELISA. Briefly, Costar 3690 96-well ELISA plates were coated with 0.5 µg/well (ovine Prt; Caslo, Denmark) overnight at 4°C, followed by a three time wash and then blocked with 100 µL/well of 5% (w/v) skim milk (Difco, USA) in PBS containing 0.05% (v/v) Tween-20 (PBS-T), for 1 h at room temperature. The ELISA plates were washed three times with PBS-T and the serum from immunized mice and from non-immunized control mouse were incubated for 1 h at 37°C, followed by another three time wash.

Alkaline phosphatase (AP)-conjugated goat anti-mouse IgG antibody (Sigma-Aldrich, St Louis, USA), diluted 1∶5000 in PBS-T, was added (100 µL/well) and incubated for 1 h at 37°C. Analysis of the AP activity (colour development) was read on an ELISA plate reader (Bio-Rad, USA) at a wavelength of 405 nm, after adding and incubating with p-Nitrophenyl Phosphate (p-NPP; J.T. Baker, The Netherlands), for 30 min at room temperature.

### 3. Western blot assay

#### 3.1. Preparation of sperm extract

Fresh ejaculated ram semen collected with an artificial vagina was used in this study. Semen was brought to the laboratory and centrifuged at 800×g for 10 min at 4°C to separate spermatozoa from seminal plasma. The sperm pellet was washed three times in phosphate-buffered saline (PBS) at pH 7.4, as described in Kamaruddin et al. [23], and resuspended in lysis buffer containing 1% Triton X-100 (v/v) at a final concentration of 5×105 spermatozoa µl-1. The mixture was incubated O/N at 4°C. Finally, the mixture was centrifuged at 10000×g for 10 min., and the pellet was resuspended in sodium dodecyl sulphate (SDS) sample buffer [24], boiled for 5 min and stored at −80°C until further use.

#### 3.2. Blocking with immunizing peptide

Demonstration of antibody specificity was carried out by antigen blocking. Briefly, 4 µl of APPA (anti-ovine Prt specific polyclonal antiserum), was incubated (37°C, 2 h) with a Prt peptide solution (20 µg/200 µl PBS-T). After centrifugation (10000×*g* for 15 min at 4°C), the supernatant was carefully removed. Total volume (2 ml) was completed with PBS-T, to reach a 1/500 dilution of the primary antibody (APPA).

#### 3.3. Western blotting

Each well was loaded with 20 µl of sperm extract (obtained from sperm pellet). Proteins were separated by SDS-PAGE electrophoresis and electroblotted onto Immobilon-PSQ Transfer Membranes (Millipore, USA) at 200 mA for 1 h (Mini-PROTEAN Tetra Electrophoresis System, Biorad). Membranes were blocked overnight at 4°C with PBS-0.2% Tween 20 buffer, containing 5% skimmed milk. Membranes were then incubated with APPA (with and without previous peptide blocking, both at a dilution of 1/500), overnight at 4°C, washed with PBS-0.2% Tween 20 buffer, and incubated for 1 h with goat anti-mouse-HRP (horseradish peroxidase enzyme conjugated) antibody (1/5000, BioRad, Cat#170-6516). The membranes were finally washed with PBS-0.2% Tween 20 buffer for 50 min and visualized with Immobilon Western Chemiluminescent HRP Substrate (Millipore, USA).

### 4. Sample collection and evaluation

#### 4.1. Collection of tissue samples

Ram testes were obtained in a slaughterhouse, from 4–6 years-old Merino sheep breed animals. Testicles were cut in pieces and fixed in buffered isoosmotic 4% (w/v) formaldehyde solution.

#### 4.2. Semen collection

Semen collection was conducted at the experimental farm of INRB in compliance with the requirements of the European Union for farm animal's welfare and the Portuguese authority guidelines for animal experimentation. Semen ejaculates were collected using an artificial vagina from three 2–6 years-old rams, two of them belonging to the Portuguese native Churra Galega Mirandesa breed of sheep and previously genotyped for the synonymous substitution (78G>A), at the codon 26 of the *prnd* gene (GA and GG genotypes) [25], and the other one belonging to the Merino breed. Semen ejaculates were also obtained from two male goats (same age) belonging to the Portuguese native Serrano goat breed. These animals were already identified by their good *in vivo* and *in vitro* fertility results. After collection, each ejaculate was immediately evaluated for volume, motility and concentration. In addition, spz viability and abnormalities (nigrosin-eosin staining) were analyzed.

#### 4.3. Preparation and evaluation of capacitated semen

After collection, ram ejaculates were kept at room temperature and light protected for up to 2 h, then washed in synthetic oviductal fluid (SOF) and centrifuged at 225×g for 5 minutes as previously described [26]. The medium used for washing the spz was SOF enriched with bovine serum albumin (4 mg mL^−1^ BSA, Sigma-Aldrich, St Louis, USA) plus glutamine (1.5 µg mL^−1^, Sigma-Aldrich, St Louis, USA). Volumes of 25 µl of the centrifuged semen were layered under 1.2 mL of capacitation medium in glass tubes and the motile spz were allowed to swim up into the medium during an incubation period of 1 hour. The upper regions of the overlays were harvested and after centrifugation at 225×g for 5 minutes, the supernatant was rejected and the remaining pellet of spz evaluated for individual motility, concentration and vigor [25].

### 5. Immunolocalization

#### 5.1. Indirect immunofluorescence staining and confocal microscopy

Ovine and caprine fresh sperm were suspended in PBS, adjusting the cell concentration to 35 to 50×10^6^ cells mL^−1^. Fifteen µL of fresh sperm suspension or of capacitated spz were smeared and air-dried on thoroughly cleaned glass slides, and smear was allowed to attach for 10 min. Slides were fixed for 5 min in absolute methanol, air dried, and kept at −20°C for further studies. Slides were washed three times for 10 min with TBS and incubated in TBS-T (TBS containing 0.1% (v/v) Tween-20) supplemented with 5% (w/v) BSA (Carl Roth GmbH+Co.KG, Germany) for 60 min, to block nonspecific sites. After blocking, slides were incubated (120 min at 4°C) either with the APPA primary antibody, with mouse control serum (pre-immune) or with TBS (negative controls). Afterwards, wells were extensively washed with TBS-T and further incubated for 60 min at room temperature with the appropriate secondary antibody, consisting of a Goat anti-Mouse IgG (gamma) antibody FITC-conjugated (Invitrogen, USA). After 30 min with the secondary antibody, 5 µl of DAPI (4,6-diamidino-2-phenylindole, dihydrochloride; Invitrogen; diluted 1/1000 in T-TBS), were added to the slide for nucleic acid staining, and allowed to incubate another 30 minutes. Finally, slides were thoroughly washed with TBS-T and images were acquired with a Zeiss LSM 510 META inverted laser scanning confocal microscope using a Plan-Apochromat 63×/1.4 objective. DAPI fluorescence was detected using a Diode 405 nm laser (50 mW nominal output) and a BP 420–480 filter. FITC fluorescence was detected using the 488 nm laser line of an Argon laser (45 mW) and a LP 505 filter. A transmitted light PMT was also used with 488 nm laser excitation for transmitted light imaging. Images were acquired with a pixel width of 140 nm (frame size 1024×1024) and pixel time 1.60 us, using sequential multi-track imaging to avoid any potential bleed-through between DAPI and FITC channels.

#### 5.2. Immunohistochemistry

Ram testicular samples fixed in 4% (w/v) formaldehyde were embedded in paraffin. Sections (4 µm) were cleared, rehydrated, blocked (endogenous peroxidase activity), incubated with the primary (APPA, diluted 1∶500 in antibody diluent reagent solution, Invitrogen) and secondary (horseradish peroxidase-conjugated anti-mouse) antibodies, treated with diaminobenzidine, and counterstained with Hematoxylin, according to the BenchMark ULTRA IHC/ISH Module staining protocol (Ventana Medical Systems, Inc.; Roche, USA). Negative controls were performed either by incubating sections with the secondary antibody alone, or using mouse control serum (pre-immune), as the primary antibody. To precisely determine the different cellular types present at the seminiferous tubules, and thus facilitate the identification of the cells expressing Prt, two other antibodies were used: one directed against Vimentin (Monoclonal Mouse Anti-Vimentin, clone Vim 3B4, Code M7020, Dako, Dilution 1/200), and the other against Ki-67 (Monoclonal Mouse Anti-Human Ki-67 antigen, clone MIB-1, Code M7240, Dako, Dilution 1/100), to locate respectively the Sertoli cell cytoskeleton, and the germinal cells with particular emphasis to the spermatides, as Ki-67 is a nuclear protein that is mainly expressed in proliferating cells [27]. Microphotographs were captured using a digital camera (Nikon sight Ds-Fi 1) mounted on a triocular optical microscope (Nikon eclipse 50 i).

### 6. Comparison between ovine and caprine Prt

#### 6.1. Sequence Alignment

Alignment of ovine and caprine Prt sequence was undertaken with the M-Coffee web server [28], a meta-method for computing multiple sequence alignments (MSAs) by combining alternative alignment methods.

#### 6.2. Amyloid propensity prediction

It has been shown that certain protein sequences have an increased propensity to ‘aggregate’ into amyloid structures resulting in a wide range of amyloid diseases. Prions are a special case among amyloid proteins because of their unusual properties. They originate from the conversion of a normal host protein into a fibrillar structure that then acts as an infectious particle [29]. Amyloidogenic propensity prediction from sequence was conducted simultaneously with different tools, as described in http://biophysics.biol.uoa.gr/AMYLPRED.

#### 6.3. Predicted secondary and tertiary structure

Both ovine (Accession n° ABO86196.1) and caprine (Accession n° CAL85353.1) Prt secondary and tertiary structures were predicted with the I-TASSER software [30], [31].

## Results

### 1. Comparison of the ovine and caprine Prt

#### 1.1. Sequence Alignment

**Figure 1:**
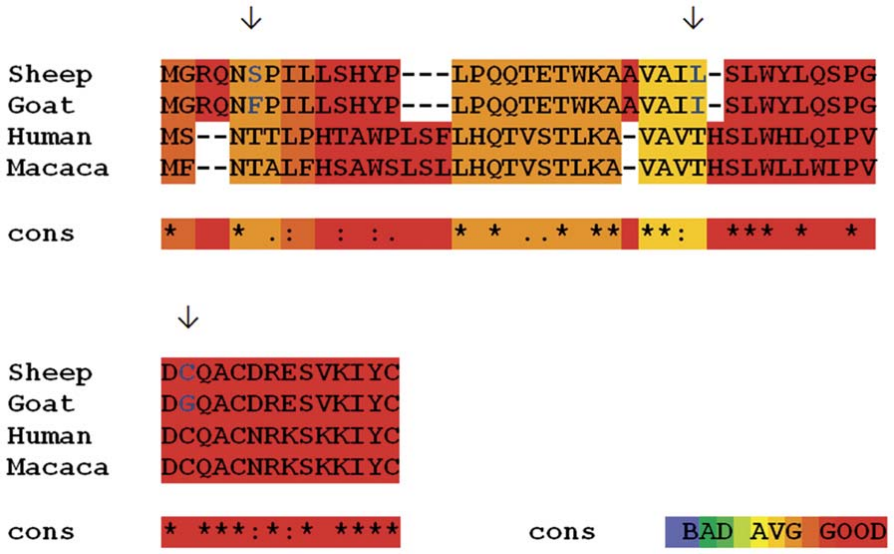
Sequence alignment of the amino acid sequences of the ovine, caprine, human and monkey Prt (prion protein testis-specific). Top lane: complete ovine Prt sequence GenBank: ABO86196.1. Second lane: complete caprine Prt sequence, GenBank: CAL85353.1. Third lane: truncated (42–94 a.a.) human Prt sequence, UniProtKB/Swiss-Prot: Q86SH4.1. Forth lane: truncated (42–94 a.a.) Rhesus monkey (*Macaca mulatta*) Prt sequence, NCBI Reference Sequence: XP_001112273.2. Blue letters with arrows on top: different amino acids between sheep and goat Prt; Asterisk (*) denotes identical residue; colon (:), conservative change; period (.), related substitution. Dark red indicates residues aligned in a similar fashion among all the individual MSAs; Dark yellow, orange and red residues can be considered to be reliably aligned. Alignment using the M-Coffee program [28].

Kocer et al. [18] demonstrated the occurrence of *prnt*-related sequences in ruminant genomes, and revealed the presence of a premature stop codon on the bovine and caprine ORF, responsible for the size difference between ruminant Prt and its human counterpart (94 aa), and a second AUG initiation codon located downstream with a G located in position +4, as described in Kozak [32] . Comparison between published ovine (ABO86196.1.) and caprine (CAL85353.1.) Prt sequences is presented in Figure 1, showing a conserved aa substitution [Leucine (L) → Isoleucine (I)], and two non-conserved substitutions [Cysteine (C) → Glycine (G), and Serine (S) → Phenylalanine (F)]. Both proteins exhibit a theoretical isoelectric point (pI) of 6.51, and a molecular weight (MW) around 5,9 kDa (ExPASy Compute pI/Mw tool). Comparison between ruminant (ovine and caprine) Prt, and primate (human and monkey) N-terminally truncated homologous protein (Fig. 1), denotes a high level of conservation between these proteins, with a pronounced homology in the terminal region.

#### 1.2. Amyloid propensity prediction for ovine and caprine Prt

**Figure 2:**
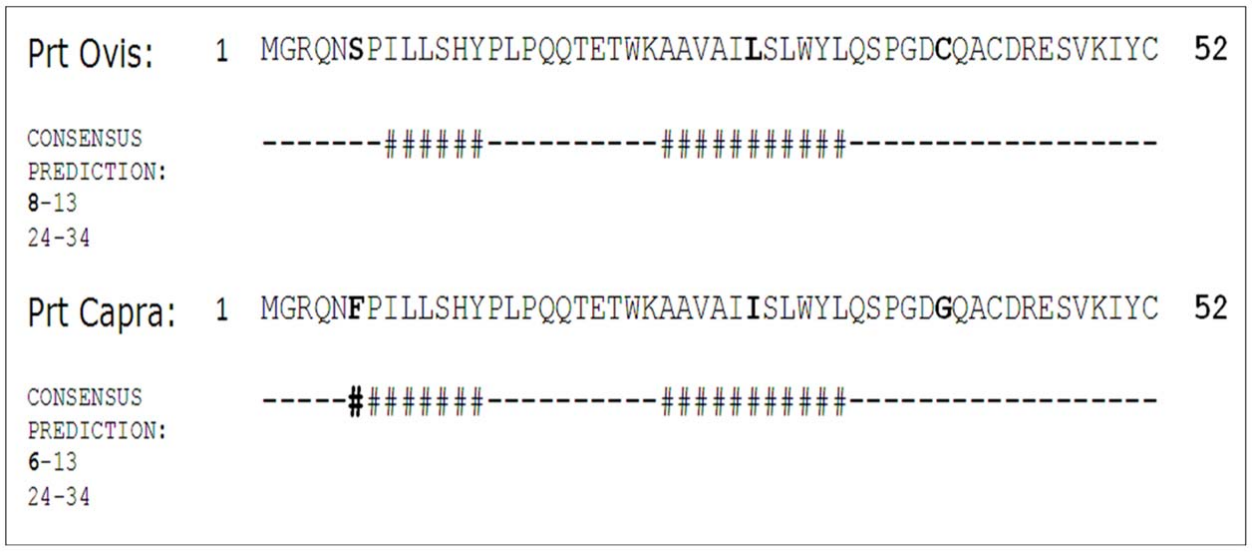
Amyloid propensity prediction for ovine and caprine prion protein testis specific (Prt). Top lane: ovine Prt sequence ABO86196.1. Second lane: caprine Prt sequence CAL85353.1. Consensus predictions for amyloid propensity [ovine (8–13 and 24–34 residues) and caprine (6–13 and 24–34 residues)] with different methods (as described in http://biophysics.biol.uoa.gr/AMYLPRED) are labeled with a number sign symbol (#). Note the key role of phenylalanine residue (caprine aa position 6) in increased amyloid formation propensity.

It is becoming increasingly clear that specific regions of the amino acid sequences of polypeptide chains, sometimes known as “aggregation-prone” regions, play a major role in determining their tendency to aggregate and, ultimately, to form organized structures such as amyloid fibrils [33]. At neutral pH, the phenylalanine residue, in caprine Prt sequence, have one of the highest amyloid aggregation propensities (agg  = 2.8), in contrast to the Serine residue, in ovine Prt sequence, found is the same sequence position (agg = −5.08), as described in [34] (Fig. 2).

#### 1.3. Predicted secondary and tertiary structure of ovine and caprine Prt

**Figure 3:**
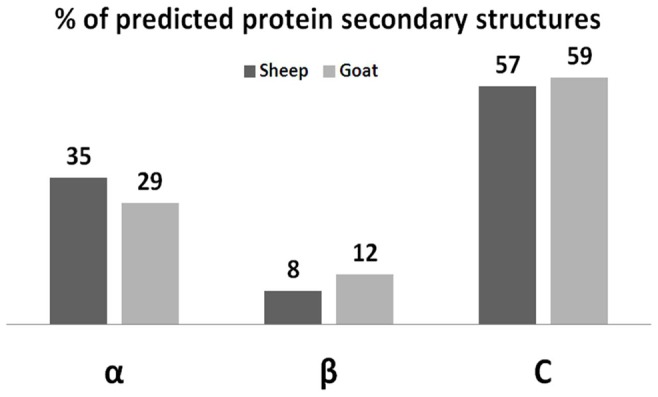
Predicted secondary structure comparison of the ovine and caprine Prt (prion protein testis-specific) proteins. α: Alpha helix; β: Beta sheet; C: Coil. I-TASSER software [30], [31].

**Figure 4:**
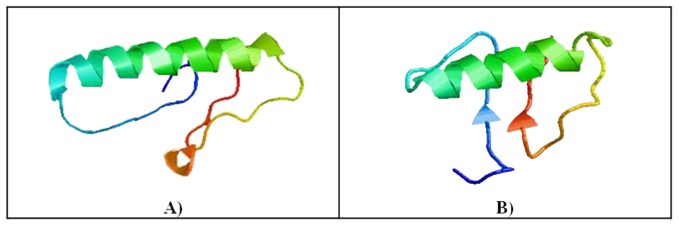
Predicted tertiary structure of: A) ovine and B) caprine prion protein testis specific (Prt), using the I-TASSER software [**30]**, [31].

Like PrP^C^, Dpl is a GPI anchored glycoprotein, structured by three α-helices and two β-sheets [35]. However, Doppel does not exhibit the *α*→*β* transformation that occurs between the normal cellular isoform, PrP^C^, and PrP^Sc^ [36]. The supersecondary structure of amyloids and prions is difficult to determine, although probability-based prediction of discrete β-strand pairs can offer insight into these structures [37]. The secondary and tertiary structures of ovine and caprine Prt predicted with the I-TASSER software [30], [31] are shown in Figures 3 and 4. In both cases, the theoretical globular model predicts a secondary structure constituted mainly by α-helixes, and with a less tendency for β-sheet strands. Note that intrinsic secondary structure propensity of residues (like phenylalanine, a large aromatic which favors β-strands) can determine to some extent the global tertiary structural features [38].

### 2. Detection of ovine Prt by Western blot, immunofluorescence and immunohistochemistry

#### 2.1. Detection of ovine Prt by Western blot

**Figure 5:**
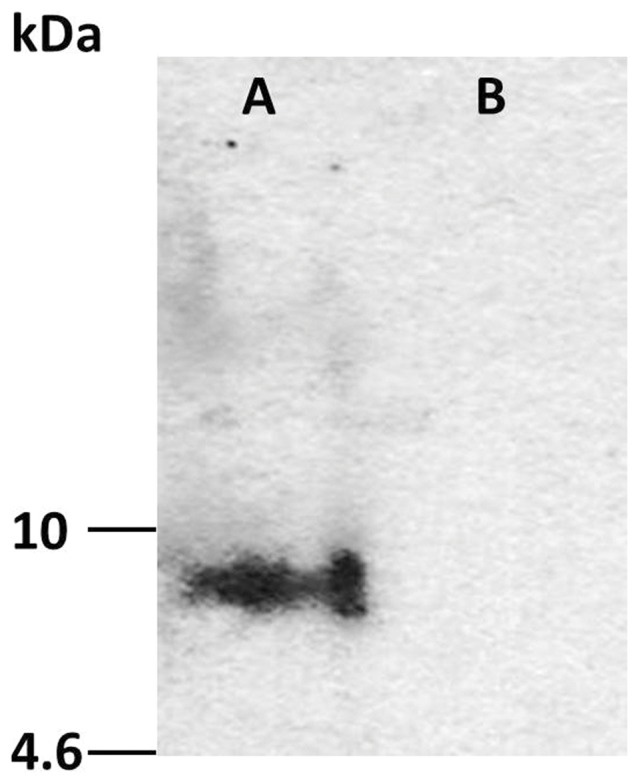
Western blot analysis. Western blot analysis of crude preparations of adult ovine spermatozoa (sperm extracts) with the anti-ovine prion protein testis specific (Prt) polyclonal antiserum (A). No hybridization was detected when the primary antibody was preabsorbed with the immunizing peptide (B). Molecular masses are indicated.

Western blot analysis revealed Prt-immunoreactivity confined to a band of about 7 kDa (Fig. 5).

#### 2.2. Location of Prt on ovine and caprine spermatozoa by immunofluorescence

After the initial evaluation, only goat or ram ejaculates of good quality (mass motility >4; individual motility >60%; concentration >2.5×10^9^ spz mL^−1^) were used for Prt location. Moreover following ovine semen capacitation, standard values were obtained for the spz individual motility, concentration and vigor [25].

**Figure 6:**
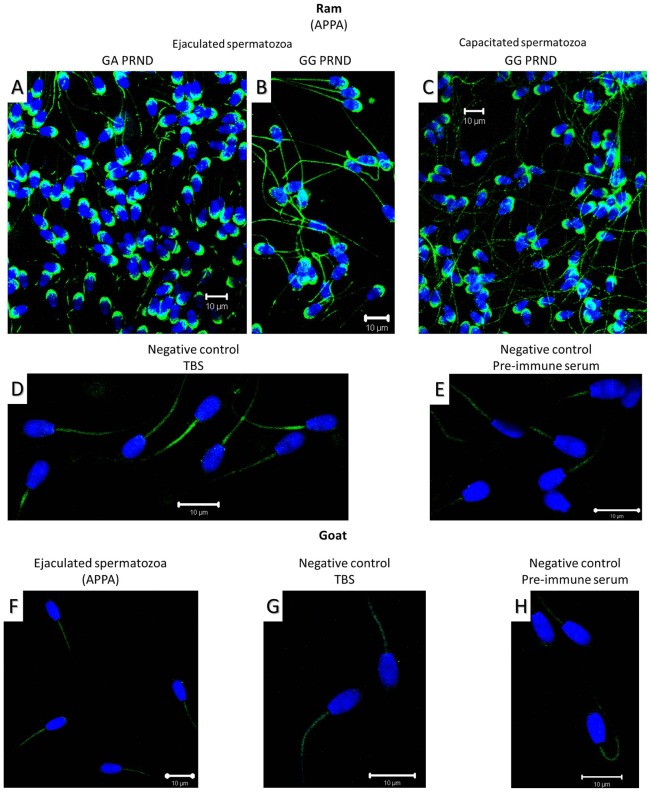
Immunofluorescence images of prion protein testis specific (Prt) location in spermatozoa, using mouse anti-ovine Prt polyclonal antibody (APPA). Ovine Prt (Churra breed) is present on the ram spermatozoon head apical ridge subdomain, corresponding to the membranes over the acrosome, after ejaculation (A and B) and *in vitro* capacitation (C). Same results were obtained for the Merino breed animals (data not shown). No immunofluorescence staining was detected with APPA on goat (Serrana breed) spermatozoa (F). Negative controls (D, E, G and H) were carried out with samples processed exactly the same way but using either pre-immune serum from the same BALB/c mouse (E and H), or TBS (D and G) as the primary antibody. Anti-mouse FITC antibody (green), nucleus (DAPI/blue), Scale bar: 10 µm.

Ovine Prt was detected by immunofluorescence, at the sperm head apical ridge subdomain of ejaculated ram spz (corresponding to the acrosome region) and after *in vitro* capacitation. Results obtained suggest that no Prt positivity was detected at the acrosome region of goat spz (Fig. 6).

#### 2.3. Detection of ovine Prt in the ram testis by immunohistochemistry

**Figure 7:**
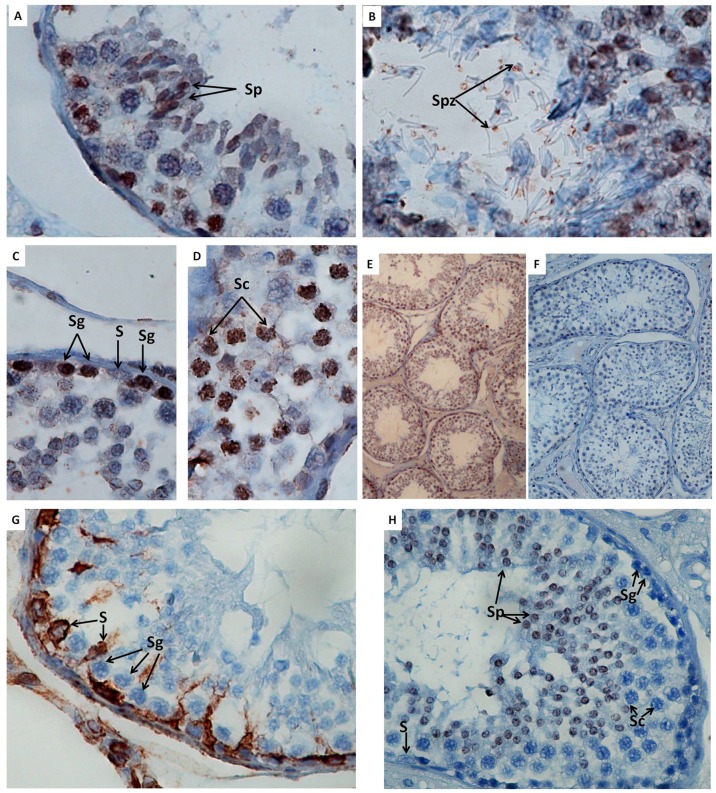
Ovine prion protein testis specific (Prt) distribution in ram testis using immunohistochemistry, and produced anti-ovine Prt polyclonal antibody (APPA). APPA labeling of ram germinal cells in seminiferous tubules (E). Positive cells were detected (arrows) in spermatogonia (C), spermatocytes (D), spermatides (A), and spermatozoa (B). Negative control using mouse control serum (pre-immune), as the primary antibody (F). Same results were obtained when incubating sections with the secondary antibody alone (data not shown). Vimentin (G) and Ki-67 antigen (H), immunodetection of Sertoli cells cytoskeletal and spermatides, respectively. Spermatogonia (Sg), spermatocytes (Sc), spermatides (Sp), spermatozoa (Spz), Sertoli cell (S). Magnifications (A, B, C and D) ×1000, (E and F) ×100.

In order to study the localization of Prt in ovine tissues, immunohistochemical assays were performed on ram testicular sections. The seminiferous epithelium undergoes cyclic changes characterized by certain cellular compositions reappearing at regular intervals [39]. Results showed that ovine Prt was found in seminiferous tubules, along the developing stages of germinal cells, but not in the wall of the spermatogenic epithelium (Fig. 7E). With a higher magnification, ovine Prt was detected in spermatogonia, spermatocytes and spermatides (Fig. 7A, 7C and 7D) and also in spz (Fig. 7B). The specificity of the staining was supported by the negative control sections obtained with the secondary antibody alone and with pre-immune serum (Fig. 7F).

## Discussion

Although the normal physiological function of prion proteins remains largely unclear, recent studies strongly suggest that some, like Doppel and Prt, seem to play a major role in the reproduction field [1], [17]. Doppel is expressed on adult testis tissue and takes an important role in maintaining sperm integrity, normal fertility and motion ability [40]. Females lacking Doppel are viable and fertile but males without Doppel are sterile. Male *prnd* knockout mice had normal sexual behavior with reduced or normal sperm concentrations but its spz were unable to perform the acrosome reaction and fertilize the oocyte [15], [16]. Moreover the localization of Doppel on both Sertoli and germ cells [41], [42] strongly suggests that this protein may play a major role in male fertility [25]. *prnd* and *prnt* are located in close proximity and in opposite orientations, a structural arrangement frequently encountered in genomic regions that have a similar structural organization and expression profiles [7].

Allied to the described expression of Prt on testis tissue [7], [18], this study intended to elucidate if this protein could also exert a key role in male fertility. Based on immunofluorescence and immunohistochemistry assays, using a newly developed anti-ovine Prt specific polyclonal antiserum (APPA), we report here for the first time the location of ovine Prt protein in spz, confirming that ovine *prnt* gene is transcribed into coding RNA and effectively translated. Besides, caprine in comparison to ovine Prt sequence seems to exhibit a slightly enhanced predicted tendency to form amyloid fibrils (Fig. 2) presenting also different predicted primary (Fig. 1), secondary (Fig. 3) and tertiary structures (Fig. 4). This appears to be related to the high intrinsic amyloidogenic propensity of the phenylalanine [43] a nonpolar aromatic aa located in the caprine protein sequence (position 6), in contrast to the serine polar aa of the ovine Prt sequence (same aa position) (Fig. 2). Many studies that have examined the molecular mechanism of TSE have suggested the possibility that not only *prnp*, but also *prnd*, *prnt*, and the Ras association domain family 2 (RASSF2), are functionally associated with prion disease in humans [7], [8], [44], [45]. Further research will be required to elucidate the functional role of *prnt* and its role in prion diseases [46]. Amyloid propensity prediction is important both for rationalizing the effects of sequence changes on the aggregation of polypeptide chains and for the development of targeted strategies to combat diseases, such as TSEs, that are associated with amyloid formation [33], [43]. This new developed polyclonal antibody antiserum could also promote further study related to the potential implications of Prt at the prion protein conversion. As previously mentioned, Prt expression pattern at the mRNA level, has already been investigated in caprine and human tissues [7], [18] but, to our knowledge, the tissue distribution and localization of this protein had not yet been explored. In humans, all three *prnt* isoforms were detected only in adult testis, and were not present in any of the human fetal (11.5–16.5 week gestation) tissues analyzed, including testis [7]. In contrast, caprine *prnt* is very weakly expressed in adult testes and ovaries [18]. Data presented here revealed that ovine Prt is present in ejaculated ram sperm head apical ridge subdomain, corresponding to the acrosome region, remaining after *in vitro* capacitation (Fig. 6A, 6B and 6C), and also in germinal cells from seminiferous tubules, including spermatogonia, spermatocytes, spermatides and spz (Fig. 7). For instance, ruminant Doppel protein encoded by the *prnd* gene is expressed intensively in testis while sparsely in other organs [9], [47]. Bovine Doppel was detected by immunocytochemistry on ejaculated spz with an intense positive staining extending from the neck to the middle piece [14]. Human Doppel was found on the flagella of epididymal and mature spz, suggesting that this protein is involved in the motility of spermatozoa after being transferred to the membrane during spz maturation [41].

Previous work developed in our group [48] identified a possible association between Doppel gene polymorphism (silent polymorphism corresponding to a 78G>A synonymous substitution in codon 26), with ram semen traits/freezability and embryo production [25], [49]. Moreover, the direct involvement of Doppel protein in enhancing spz fertilizing ability was also determined [17]. With APPA, we consistently observed an intense staining of the ovine ejaculated spz acrosome, unrelated to the *prnd* genotype or to the capacitation status (Fig. 6A, 6B and 6C). Capacitation is broadly defined as the functional modifications rendering sperm competent to fertilize, encompassing the ability of the sperm to bind to the zona pellucida and subsequently undergo the acrosome reaction [50]. For instance, ovine Prt staining of the acrosome throughout the capacitation process could indicate a hypothetical involvement of this protein in the subsequent sperm-egg interaction. As with human Doppel [41], Prt immunofluorescence staining was also present on the flagella of ram spz (Fig. 6A, 6B, 6C and 6F). However, because similar patterns could also be observed (Fig. 6D, 6E, 6G and 6H) in negative controls (using either pre-immune serum or TBS, instead of the primary antibody), we concluded that spz flagella staining could possibly be the consequence of nonspecific staining from the secondary antibody. For instance, no specific staining was detected with APPA on ejaculated goat spz (Fig. 6F), which could be explained by the different predicted primary (Fig. 1), secondary (Fig. 3) and tertiary structures (Fig. 4) for the caprine and ovine Prt protein, confirming the specificity of the APPA antibody.

Choi et al. [46], concluded that *prnt* is only present in primates and not in bovine, indicating that the *prnt* gene appeared in the primate lineage after the evolutionary split from rodents. This could be explained by the low homology between the human and ovine *prnt* sequences. Kocer et al. [18] suggested either that the expression pattern of Prt differs between ruminant and human or, most probably, that ruminant *prnt* is a pseudogene. As referred, some pseudogenes appear to harbor the potential to regulate their protein-coding cousins, being transcribed into noncoding RNA that contributes to normal cellular regulation [25]. Premzl and Gamulin [2] suggested that *prnt* could be viewed as a TE (transposable element)-associated gene. Apart from proving that *prnt* is a protein-coding gene, we managed to detect ovine Prt protein, with immunohistochemistry, along the developing stages of ram germinal cells and with immunofluorescence at the ram sperm head apical ridge subdomain, corresponding to the acrosome region. During mammalian spermatogenesis, spermatogenic stem cells proliferate to produce spermatogonia, which differentiate into primary spermatocytes. After a prolonged meiotic prophase, the latter undergo two consecutive meiotic divisions to generate haploid spermatids. Spermatids then undergo marked morphological changes from an initially round to a progressively elongated shape with condensed nuclei, and eventually form spz [51]. Contrastingly to ovine Dpl [42], ovine Prt was found in seminiferous tubules, along the developing stages of germinal cells (Fig. 7), but not in the wall of the spermatogenic epithelium or at the Sertoli cells. Primarily expression in the nuclei of spermatogonia and spermatocytes, and subsequently in the elongated spermatides (Fig. 7) and in the spz acrosome (Fig. 6), unrelated to spz capacitation, indicate that ovine Prt may play an important role in ram spermatogenesis, including spermatogenic cell proliferation and sperm maturation, as well as in fertilization.

In conclusion, our data shows that ovine *prnt* is a translated protein-coding gene and not a pseudogene. The data strongly suggest a major physiological function of Prt in ovine reproduction. As an important spermatogenesis-related protein, the biochemical structure, function and role of Prt in different stages of spermatogenesis should be studied in greater detail in the near future.
